# The clinical significance of circulating GPC1 positive exosomes and its regulative miRNAs in colon cancer patients

**DOI:** 10.18632/oncotarget.20516

**Published:** 2017-08-24

**Authors:** Jian Li, Bo Li, Caiping Ren, Yuxiang Chen, Xiong Guo, Lin Zhou, Zha Peng, Yaping Tang, Yang Chen, Weidong Liu, Bin Zhu, Lei Wang, Xuxu Liu, Xiao Shi, Zixuan Peng

**Affiliations:** ^1^ Hepatobiliary and Enteric Surgery Research Center, Xiangya Hospital, Central South University, Changsha, Hunan 410008, China; ^2^ Department of Pathology, Xiangya Medical School, Central South University, Changsha, Hunan 410078, China; ^3^ Cancer Research Institute, Xiangya Hospital, Collaborative Innovation Center for Cancer Medicine, Key Laboratory for Carcinogenesis of Chinese Ministry of Health, School of Basic Medical Science, Central South University, Changsha, Hunan 410078, China; ^4^ School of Pharmaceutical Science, Central South University, Changsha, Hunan 410013, China

**Keywords:** colorectal cancer, exosome, biomarker, GPC1, miRNA

## Abstract

Colorectal cancer (CRC) is a leading cause of cancer-related deaths worldwide. Recent study found an increased level of glypican-1 positive (GPC1^+^) plasma exosomes in patients with stage II CRC, but decreased levels of plasma miR-96-5p and miR-149. This study further investigated the clinical significance of plasma GPC1^+^ exosomes and plasma miR-96-5p and miR-149 levels in stage III CRC patients. To study the effect of these microRNAs on GPC1^+^ plasma exosomes, we isolated and purified exosomes and overexpressed human GPC1 and the microRNAs miR-96-5p and miR-149 by adenovirus vectors. Overexpression of GPC1 activated epithelial-mesenchymal transition (EMT) which then increased invasion and migration in HT29 and HCT-116 colon cancer cells. In contrast, silencing GPC1 expression and overexpressing miR-96-5p and miR-149 significantly inactivated EMT and decreased invasion and migration of HT29 and HCT-116 cells. miR-96-5p and miR-149 inhibitors significantly increased invasion and migration of HT29 and HCT-116 cells. Our results indicate that high levels of circulating GPC1 positive exosomes before and after surgery as well as low circulating miR-96-5p and miR-149 before surgery indicated a severe clinical status and poor prognosis in stage III colon cancer patients. We conclude that GPC1 can be a biomarker for relapse of stage III CRC and may be involved in EMT activation, invasion, and migration of colorectal cancer cells.

## INTRODUCTION

Colorectal cancer (CRC) is one of the most common gastrointestinal cancers and the second leading cause of cancer-related deaths worldwide [1]. Surgery is the first option for resectable tumors while chemotherapy and radiotherapy are the main treatments for advanced diseases and are the adjuvant treatments for surgical therapy in CRC patients [2, 3]. Although considerable progress has been made in screening, early diagnosis, and even individualized therapy, the morbidity of CRC is still high [4]. A study on recurrence-free survival (RFS) showed that the RFS rates were 98%, 92%, 90%, and 89% in stage II colorectal cancer patients, and 94%, 78%, 70%, and 66% in stage III patients at one, three, five, and seven years following surgery [5]. However, recurrence is much higher for curative resection, as one study demonstrated a cumulative recurrence rate of 100% at 4 years in patients with curative resection [6]. Therefore, it is important to identify new biomarkers for the early detection and prediction of relapse in CRC.

Glypican-1 (GPC1) is a cell surface protein of the heparan sulfate proteoglycan family. Previous studies have observed that GPC1 is overexpressed in the tissues for pancreatic cancer, breast cancer, and glioma [7–9]. A recent study reported that GPC1 expression was significantly upregulated in human CRC tissues [10], which is consistent with our own findings [11]. Additionally, GPC1 has been demonstrated to be specifically enriched in tumor cell-derived exosomes in pancreatic cancer, and the GPC1^+^ exosome was proposed as a marker for the early diagnosis of pancreatic cancer and prediction of the prognosis in pancreatic cancer patients [12]. Our previous study also demonstrated that GPC1^+^ exosomes were significantly increased in both the plasma and the tumor tissues of CRC patients [11], however, the clinical significance of GPC1^+^ exosomes in CRC patients has not yet been elucidated.

GPC1 is involved in tumor growth and angiogenesis [7–9] through the activation of FGF-FGFR signaling [13] and GPC1 expression can be suppressed by miR-96-5p in pancreatic cancer cells [14] or regulated by miRNA-149 in human endothelial cells [15]. Our recent study revealed that low miR-96-5p and miR-149 levels correlated with high GPC1 expression in colorectal cancer cells in stage I and stage II patients [11], however, the mode of regulation of these two miRNAs and whether they directly target the micro RNAs of *GPC1* gene in colon cancer cells has not yet been addressed.

GPCs have been demonstrated to affect the binding properties and subsequent functions of fibroblast growth factor (FGF) and bone morphogenetic protein (BMP) [16]. BMPs are thought to induce endothelial mesenchymal transformation (EMT) in cancer cells through SMAD and non-SMAD signaling pathways [17], while fibroblast growth factor-2 (FGF-2) has been revealed to mediated EMT in corneal endothelial cells [18]. Moreover, FGF2 increased shedding of transmembrane proteins syndecan 1 (SDC1) can activate GPC1-dependent EMT signaling [19]. However, SDC1 was found to coexpress with E-cadherin during EMT in breast cancer cells [20]. We therefore hypothesized that GPC1 may induce EMT in CRC tumor cells.

Here, we investigate the clinical significance of the concentrations of circulating GPC1 positive exosomes, miR-96-5p, and miR-149 in 85 patients with stage III colon cancer, as well as the biological function of GPC1 in colon cancer cells. We then address whether circulating miR-96-5p and miR-149 is a potential biomarker for predicting the progression, prognosis, and relapse of colon cancer patients.

## RESULTS

### Clinical significance of GPC1^+^ plasma exosomes, and plasma miR-96-5p and miR-149

**Figure 1:**
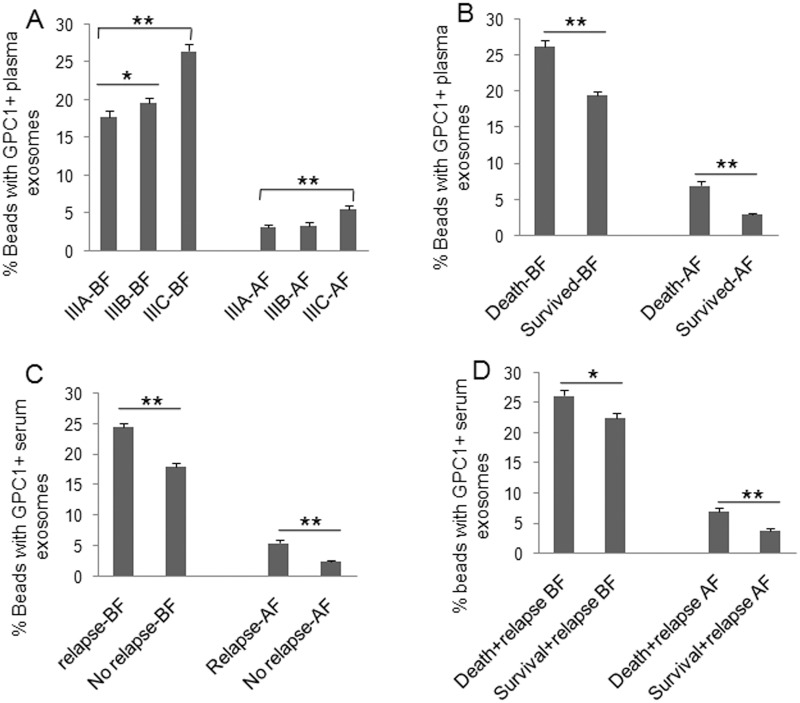
Clinical significance of plasma GPC1 positive exosomes GPC1^+^ plasma exosomes were measured using cytometry assay. The percentage of GPC1^+^ plasma exosomes in colon cancer patients was calculated. **(A)** Comparison of the percentage of GPC1^+^ plasma exosomes at one day before (BF) and one week after surgery (AF) between patients with IIIA (n=25), IIIB (n=32), and IIIC (n=28) clinical stage disease. **(B)** Comparison of the percentage of GPC1^+^ plasma exosomes at one day before and one week after surgery between died patients (n=24) and survived patients (n=61) in 2 years of followed-up. **(C)** Comparison of the percentage of GPC1^+^ plasma exosomes at one day before and one week after surgery between patients with relapse (n=44) and patients without relapse (n=41) during the 2-year follow-up. **(D)** Comparison of the percentage of GPC1^+^ plasma exosomes at one day before and one week after surgery between died patients (n=24) and survived patients (n=20) without relapse during the 2-year follow-up. ^*^P<0.01, ^**^P<0.001 between two groups.

**Figure 2:**
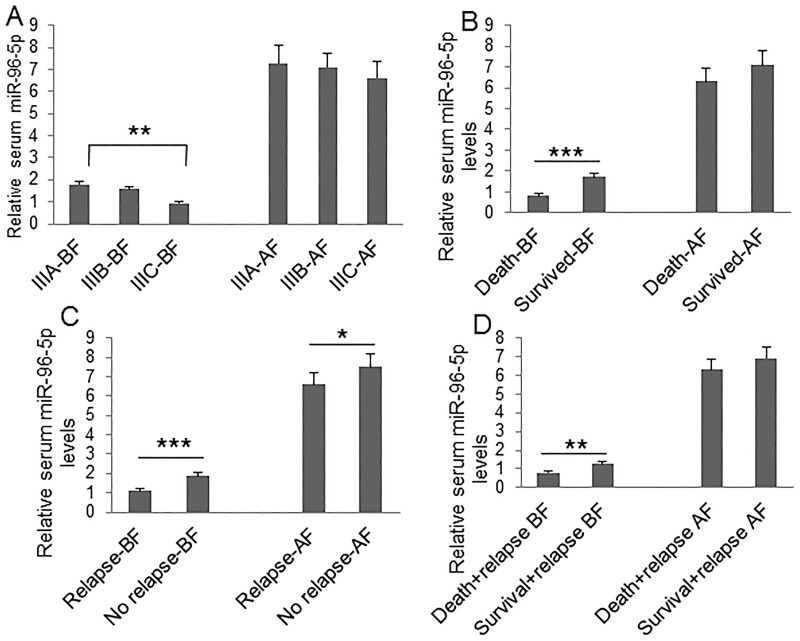
Clinical significance of plasma miR-96-5p level Plasma miR-96-5p was measured using real-time PCR. **(A)** Comparison of plasma miR-96-5p levels at one day before (BF) and one week after surgery (AF) between patients with IIIA (n=25), IIIB (n=32), and IIIC (n=28) clinical stage disease. **(B)** Comparison of plasma miR-96-5p levels at one day before and one week after surgery between died patients (n=24) and survived patients (n=61) during 2-year followed-up before and after surgery. **(C)** Comparison of plasma miR-96-5p levels at one day before and one week after surgery between patients with relapse (n=44) and patients without relapse (n=41) during the 2-year follow-up. **(D)** Comparison of plasma miR-96-5p levels at one day before and one week after surgery between died patients (n=24) and survived patients (n=20) without relapse during the 2-year follow-up. ^*^P<0.05, ^**^P<0.01, ^***^P<0.001 between two groups.

**Figure 3:**
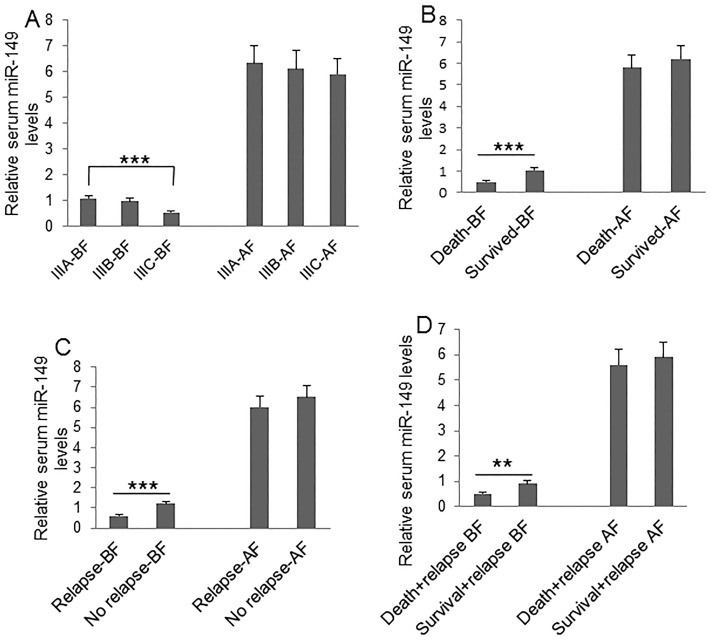
Clinical significance of plasma miR-149 level Plasma miR-149 was measured using real-time PCR. **(A)** Comparison of plasma miR-149 levels at one day before (BF) and one week after surgery (AF) between patients with IIIA (n=25), IIIB (n=32), and IIIC (n=28) clinical stage disease. **(B)** Comparison of plasma miR-149 levels at one day before and one week after surgery between died patients (n=24) and survived patients (n=61) during 2-year followed-up before and after surgery. **(C)** Comparison of plasma miR-149 levels at one day before and one week after surgery between patients with relapse (n=44) and patients without relapse (n=41) during the 2-year follow-up. **(D)** Comparison of plasma miR-149 levels at one day before and one week after surgery between died patients (n=24) and survived patients (n=20) without relapse during the 2-year follow-up. ^*^P<0.05, ^**^P<0.01, ^***^P<0.001 between two groups.

To understand the clinical significance of GPC1^+^ plasma exosomes, miR-96-5p, and miR-149, we measured their abundance in colon cancer patients before and after surgery. Plasma GPC1^+^ exosomes were measured by a cytometry assay and micro RNAs were measured using real-time PCR. The percentage of GPC1^+^ plasma exosomes one day before and one week after surgery was significantly higher in colon cancer patients with IIIC stage disease than that in patients with IIIA stage disease (Figure 1A). We also observed higher levels of GPC1^+^ plasma exosomes in several other cases: in patients that died within two years versus patients that survived over two years (Figure 1B), in patients with relapse versus patients without relapse (Figure 1C), and in patients that died with relapse versus patients that survived with relapse (Figure 1D). We observed that the plasma miR-96-5p levels were significantly lower in similar cases, such as: one day before surgery in IIIC stage colon cancer patients versus IIIA stage patients (Figure 2A), in patients that died within two years than patients versus patients that survived over two years (Figure 2B), one day before and one week after surgery for patients with relapse versus patients without relapse (Figure 2C), and also in patients that died with relapse versus patients that survived with relapse (Figure 2D). The plasma miR-149 levels were significantly lower in the corresponding cases: one day before surgery but not one week after surgery in the patients with IIIC stage colon cancer versus the patients with stage IIIA CRC (Figure 3A), in the patients that died within two years versus patients that survived over two years (Figure 3B), in the patients with relapse than versus patients without relapse (Figure 3C), and in that patients died with relapse versus patients that survived with relapse (Figure 3D).

### Short overall survival and relapse is correlated with high GPC1^+^ plasma exosomes, low plasma miR-96-5p, and low plasma miR-149 levels

**Figure 4:**
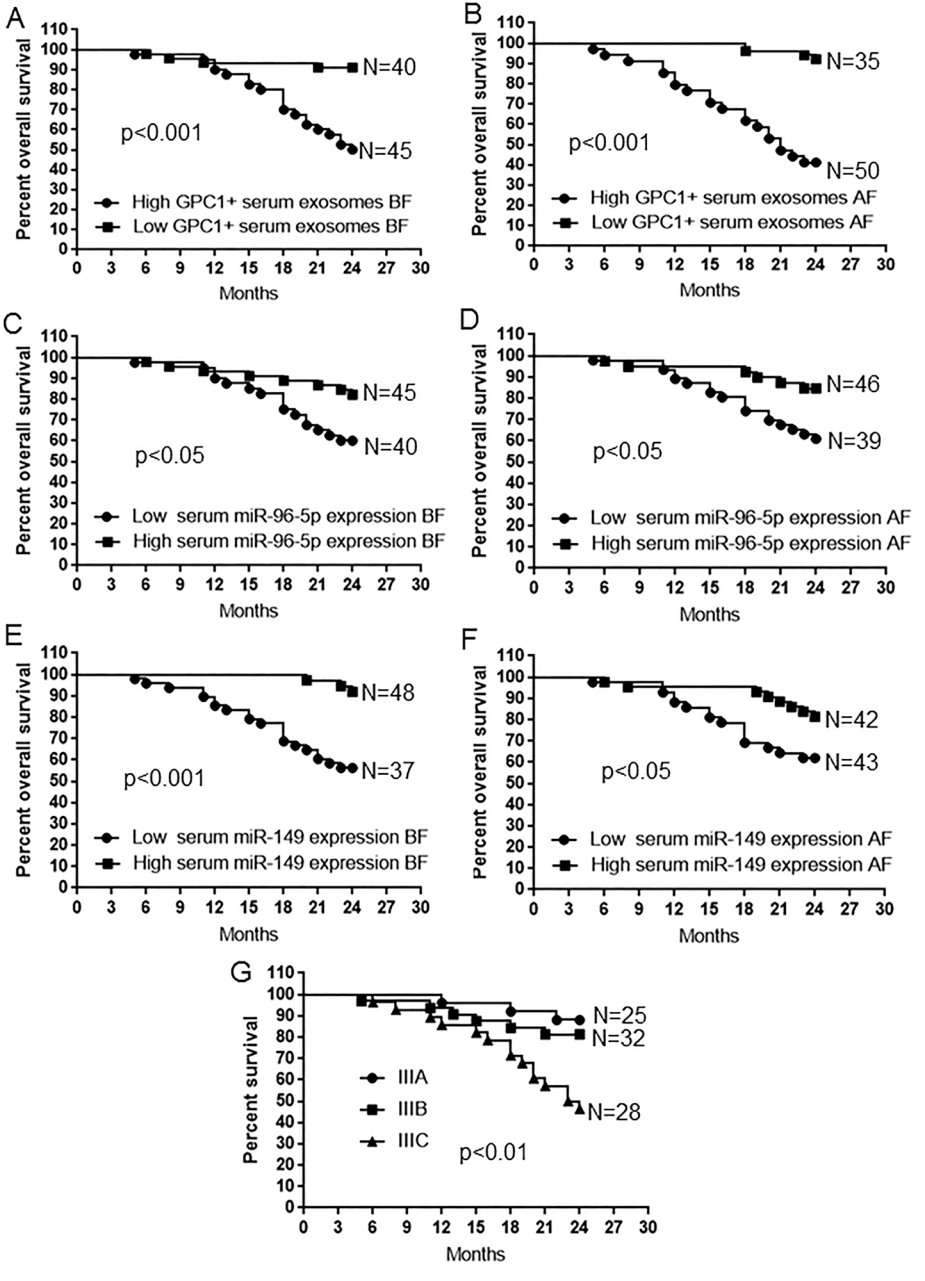
Survival analysis in patients with colon cancer **(A-B)** Kaplan-Meier plots of overall survival in patients with colon cancer and high and low GPC1^+^ plasma exosomes one day before (BF) (A) and one week after surgery (BF) (B). **(C-D)** Kaplan-Meier plots of overall survival in patients with colon cancer and high and low plasma miR-96-5p levels one day before (BF) (C) and one week after surgery (AF) (D). **(E-F)** Kaplan-Meier plots of overall survival in patients with colon cancer and high and low plasma miR-149 levels one day before surgery (BF) (E) and one week after surgery (AF) (F). **(G)** Kaplan-Meier plots of overall survival in patients with colon cancer and IIIA, IIIB, and IIIC stage disease.

**Figure 5:**
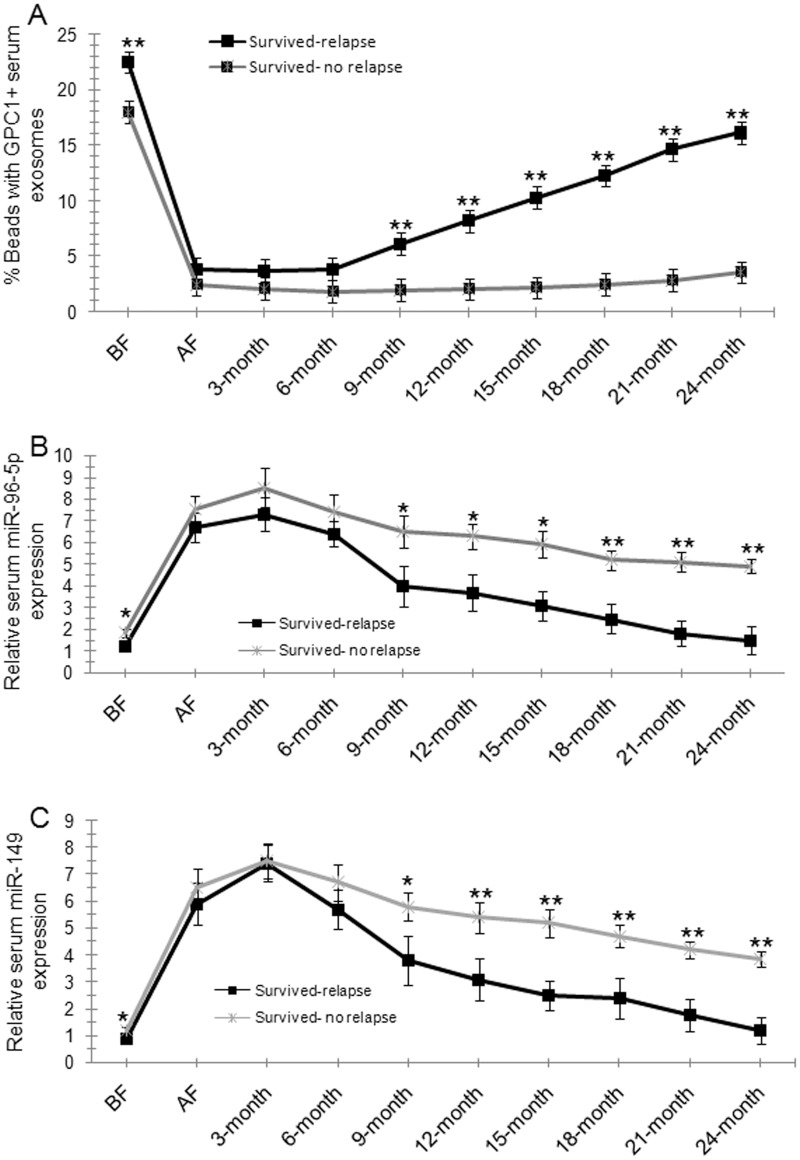
Dynamic observations of percentage GPC1^+^ plasma exosomes, plasma miR-96-5p, and plasma miR-149 levels in colon cancer patients **(A)** The percentage of GPC1^+^ plasma exosomes in survived colon cancer patients with or without relapse during the 2-year follow-up. BF: one day before surgery. AF: one week after surgery. 3-month: 3 months after surgery. **(B)** Plasma miR-96-5p levels in survived colon cancer patients with or without relapse during the 2-year follow-up. **(C)** Plasma miR-149 levels in survived colon cancer patients with or without relapse during the 2-year follow-up.

Kaplan-Meier survival analysis was used to compare the mean overall survival time between CRC patients with high and low percentage of GPC1^+^ plasma exosomes, high and low plasma miR-96-5p and miR-149 levels, and different TNM stages. The “high” and “low” was defined referring to the mean of all samples. For example, “high” means a person who had a higher percentage of GPC1^+^ plasma exosomes than the mean percentage of GPC1^+^ plasma exosomes in all samples. Results showed that a short overall survival time in patients with colon cancer is significantly correlated with a high percentage of GPC1^+^ plasma exosomes one day before (Figure 4A) and one week after (Figure 4B) surgery (p<0.001), and low plasma miR-96-5p (Figure 4C, 4D) (p<0.05) and low plasma miR-149 (Figure 4E, 4F) (p<0.05, p<0.01) levels one day before (Figure 4C, 4E) and one week after (Figure 4D, 4F) surgery. The TNM stage negatively correlated with overall survival and positively correlated with mortality (Figure 4G, p<0.01). We further observe the dynamic changes of the percentage of GPC1^+^ plasma exosomes, plasma miR-96-5p, and plasma miR-149 levels. The percentage of GPC1^+^ plasma exosomes in total plasma exosomes one day before surgery and 9-24 months after surgery was significantly higher in survived colon cancer patients with relapse than in the patients without relapse (Figure 5A). The percentage of GPC1^+^ plasma exosomes progressively increased nine months after surgery in survived colon cancer patients with relapse (Figure 5A). The plasma miR-96-5p (Figure 5B) and miR-149 (Figure 5C) levels was significantly lower in survived colon cancer patients with relapse than that in the patients without relapse one day before or one week after surgery and/or 9-24 months after surgery.

### GPC1 expression in colon cancer cells is directly regulated by miR-96-5p and miR-149

**Figure 6:**
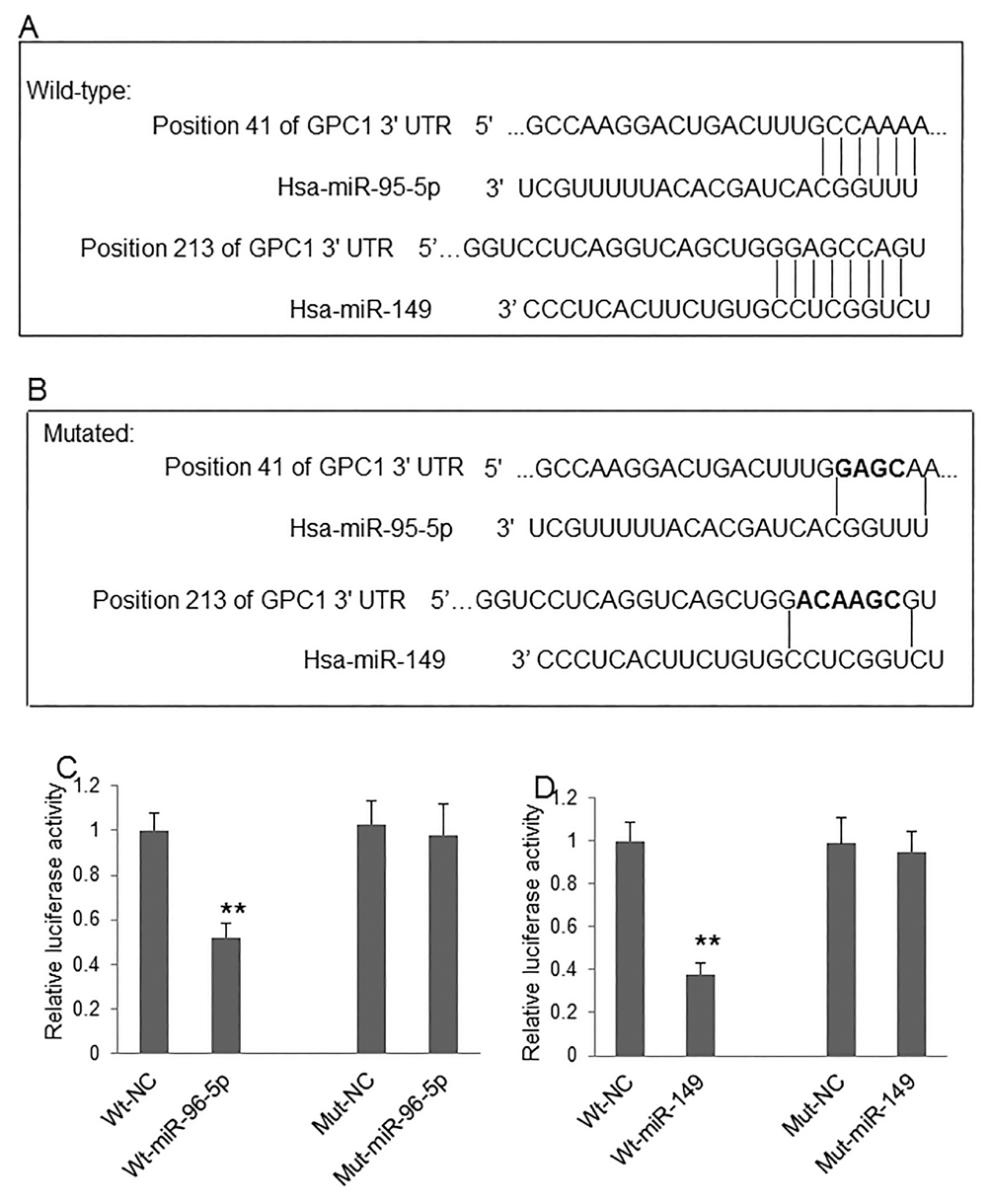
MiR-96-5p and miR-149 regulate GPC1 expression **(A)** The predicted targeting sites of miR-96-5p and miR-149 at the 3’-UTR of *GPC1* gene. **(B)** The mutated targeting sites (black) at the 3’-UTR of *GPC1* gene. **(C)** Relative luciferase activity of *GPC1* gene 3’-UTR (wild-type, Wt) and its mutation (Mut) reporter after co-infection of miR-96-5p mimics. **(D)** Relative luciferase activity of *GPC1* gene 3’-UTR and its mutation reporter after co-infection of miR-149 mimics. NC: negative control mimics. N=4. ^**^P<0.001.

Although the regulative effect of miR-96-5p and miR-149 on *GPC1* gene expression was previously implicated in colon cancer cells [11], the direct targets of these micro RNAs have not yet been identified. The direct regulation of these two miRNAs on *GPC1* gene expression, the binding sites of miR-96-5p and miR-149 on the 3’-UTR of human *GPC1* gene was predicted by bioinformatics (Figure 6A). The predicted binding sites were then mutated by overlap PCR (Figure 6B). We then used the dual-luciferase reporter assay system to investigate whether miR-96-5p (Figure 6C) and miR-149 (Figure 6D) regulate the expression of human *GPC1* gene through directly binding to the 3’-UTR sequence. Our results show that co-transfection of pmiR-RB-GPC1-3’UTR Wt plasmid with miR-96-5p or miR-149 mimics significantly decreased luciferase reporter activity in 293T cells. In contrast, co-transfection of pmiR-RB-GPC1-3’UTR Mut plasmid with miR-96-5p or miR-149 mimics showed no significant effect on luciferase reporter activity in 293T cells. These findings suggested that miR-96-5p or miR-149 directly target human *GPC1* gene at the identified targeting sequence.

### GPC1 overexpression induces epithelial-mesenchymal transition in colon cancer cells

**Figure 7:**
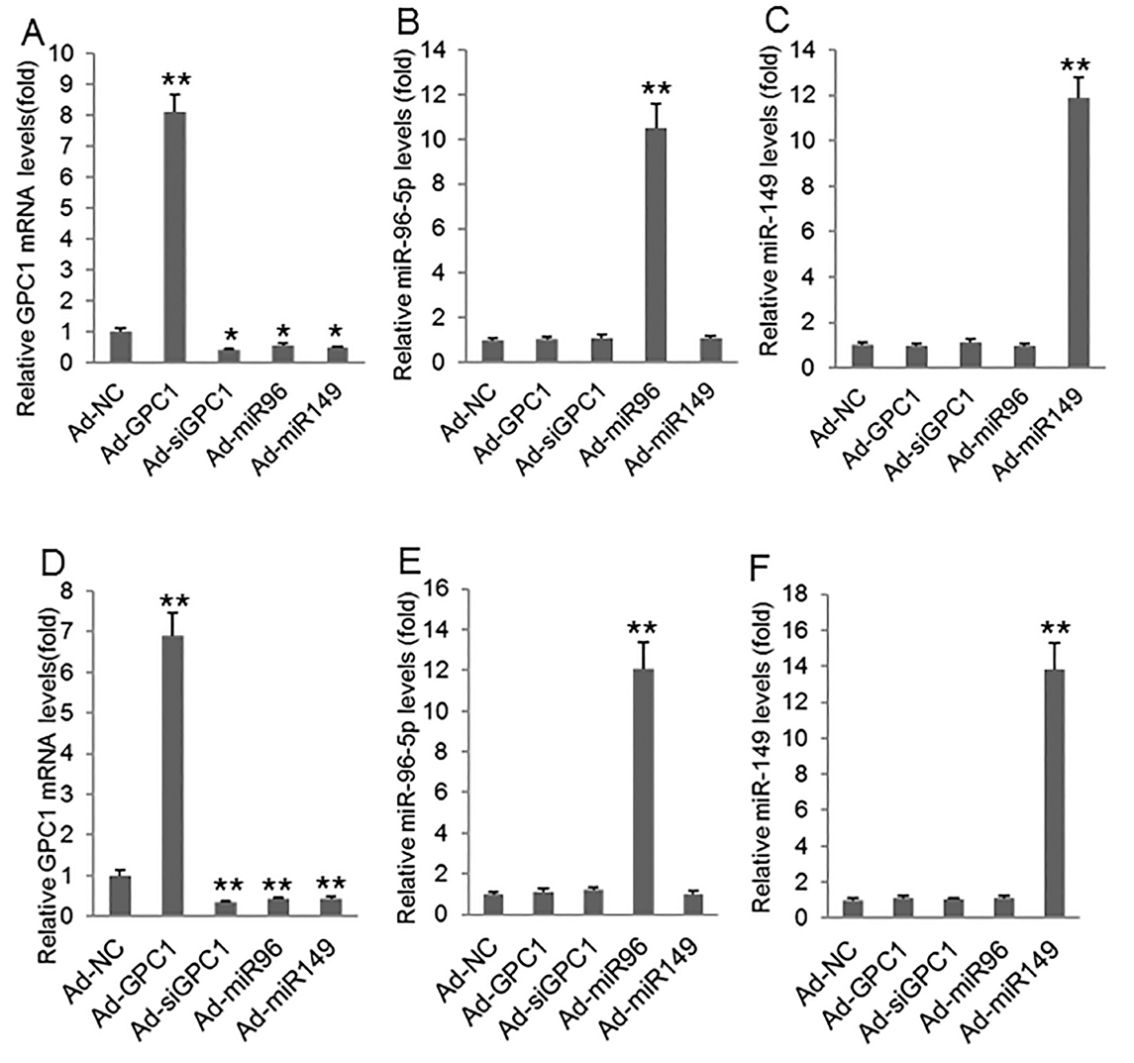
GPC1, miR-96-5p, and miR-149 mRNA levels after virus infection **(A-C)** Relative GPC1 mRNA (A), miR-96-5p (B), and miR-149 levels (C) measured by real-time PCR in HT29 cells infected with control adenovirus (Ad-NC), *GPC1* gene overexpression virus (Ad-GPC1), *GPC1* siRNA expression virus (Ad-siGPC1), miR-96-5p expression virus (Ad-miR96), and miR-149 expression virus (Ad-miR149). **(D-F)** Relative GPC1 mRNA (D), miR-96-5p (E), and miR-149 levels (F) measured by real-time PCR in HCT-116 cells infected with Ad-NC, Ad-GPC1, Ad-siGPC1, Ad-miR96, and Ad-miR149. ^*^P<0.01, ^**^P<0.001*vs.* Ad-NC. N=4.

**Figure 8:**
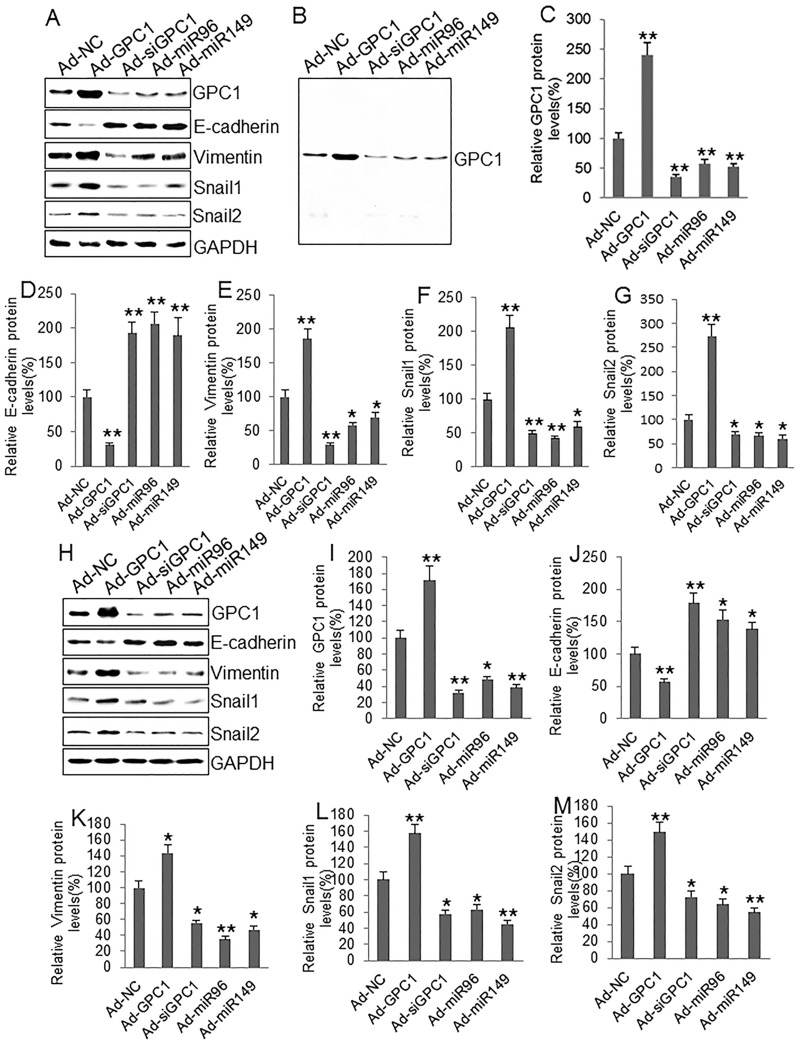
The effect of *GPC1* gene expression on EMT molecules in colon cancer cells **(A)** Representative Western blots of GPC1 and EMT-associated protein expression in HT29 cells. **(B)** a representative full-sized blot of GPC1. **(C-G)** Semi-quantitative analysis of GPC1 (C), E-cadherin (D), vimentin (E), Snail1 (F), and Snail2 (G) protein expression in HT29 cells infected with control adenovirus (Ad-NC), *GPC1* gene overexpression virus (Ad-GPC1), *GPC1* siRNA expression virus (Ad-siGPC1), miR-96-5p expression virus (Ad-miR96), and miR-149 expression virus (Ad-miR149). **(H)** Representative Western blots of GPC1 and EMT-associated protein expression in HCT-116 cells. **(I-M)** Semi-quantitative analysis of GPC1 (I), E-cadherin (J), vimentin (K), Snail1 (L), and Snail2 (M) protein expression in HT-29 cells infected with control adenoviruses described above. ^*^P<0.01, ^**^P<0.001 *vs.* Ad-NC. N=4.

To investigate the epithelial-mesenchymal transition we infected HT29 and HCT-116 cells with control adenovirus (Ad-NC), GPC1 overexpression virus (Ad-GPC1), *GPC1* siRNA expression virus (Ad-siGPC1), miR-96-5p expression virus (Ad-miR96), and miR-149 expression virus (Ad-miR149) and the protein levels of EMT biomarkers E-cadherin, vimentin, Snail1, and Snail2 [21] were measured by Western blot. HT29 (Figure 7A, 7B, 7C) and HCT-116 (Figure 7D, 7E, 7F) cells infected with Ad-GPC1, Ad-miR96, and Ad-miR149 virus significantly expressed *GPC1*, miR-96-5p, and miR-149 mRNA, respectively. Western blots showed that overexpression of *GPC1* gene expression decreased E-cadherin, but increased vimentin, Snail1, and Snail2 expression in HT29 (Figure 8A-8F) and HCT-116 (Figure 8G-8L) cells. In contrast, knockdown of *GPC1* gene expression increased E-cadherin, but decreased vimentin, Snail1, and Snail2 expression in HT29 (Figure 8A-8F) and HCT-116 (Figure 8G-8L) cells. Overexpression of miR-96-5p and miR-149 significantly increased E-cadherin, but decreased vimentin, Snail1, and Snail2 expression in HT29 (Figure 8A-8F) and HCT-116 (Figure 8G-8L) cells.

### GPC1 expression affects cell invasion and migration

**Figure 9:**
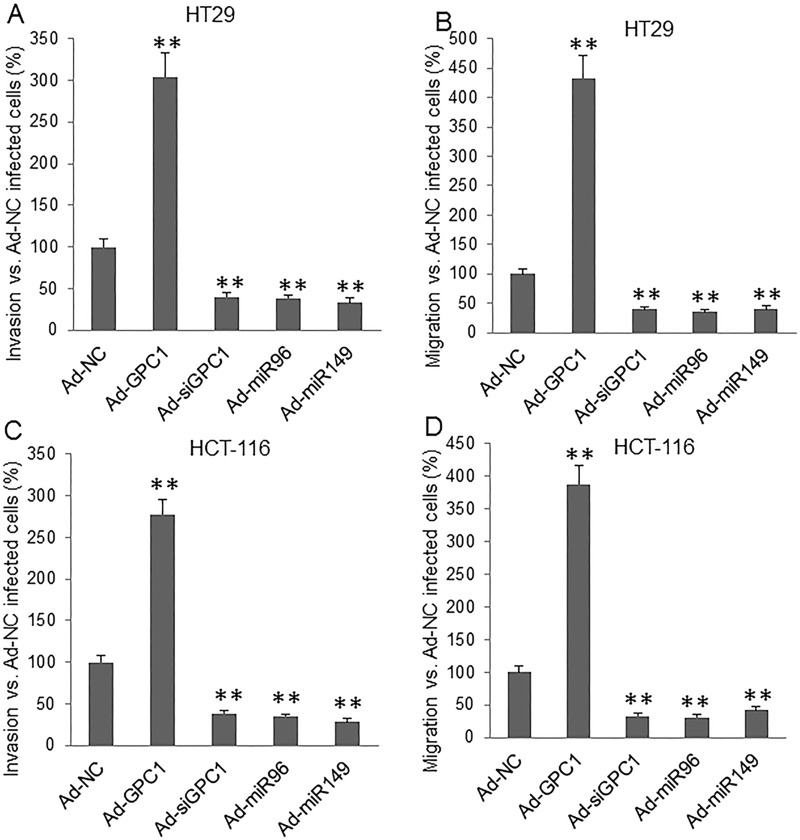
Invasion and migration assay underlying GPC1, miR-95-5p, and miR-149 overexpression and inhibition of GPC1 expression HT29 **(A, B)** and HCT-116 **(C, D)** cells were infected with control adenovirus (Ad-NC), *GPC1* gene overexpression virus (Ad-GPC1), *GPC1* siRNA expression virus (Ad-siGPC1), miR-96-5p expression virus (Ad-miR96), and miR-149 expression virus (Ad-miR149) for 12 hrs and then subjected to invasion (A, C) and migration (B, D) assay. ^**^P<0.001 *vs.* Ad-NC. N=4.

**Figure 10:**
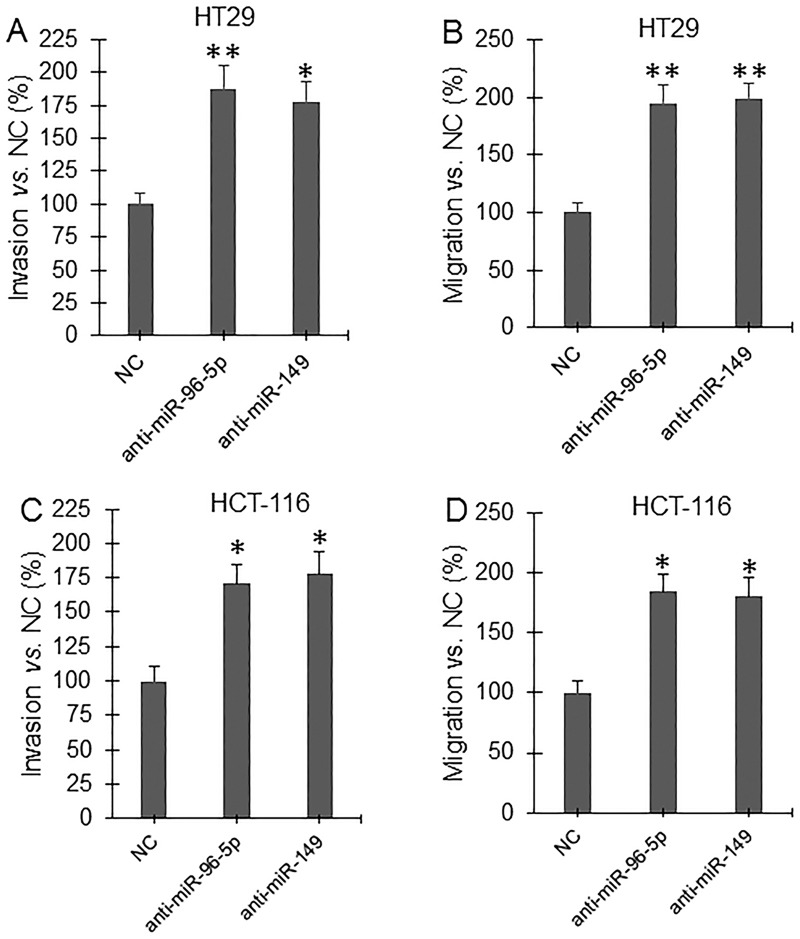
Invasion and migration assay under inhibition of miRNA expression HT29 **(A, B)** and HCT-116 **(C, D)** cells were transfected with control miRNA antisense nucleotide (NC), miR-96-5p antisense nucleotide (anti-miR-96-5p), and miR-149 antisense nucleotide (anti-miR-149) for 48 hrs and then subjected to invasion (A, C) and migration (B, D) assay. ^*^P<0.01, ^**^P<0.001 *vs.* NC. N=4.

Invasion and migration assay showed that overexpression of *GPC1* gene significantly increased, but knockdown of *GPC1* gene expression significantly decreased, and overexpression of miR-96-5p and miR-149 significantly decreased invasion (Figure 9A, 9C) and migration (Figure 9B, 9D) in HT29 (Figure 9A, 9B) and HCT-116 (Figure 9C, 9D) cells. Inhibition of miRNA expression was established by inhibitors (miRNA antisense nucleotides). Transfection of miR-96-5p and miR-149 inhibitor significantly increased invasion (Figure 10A, 10C) and migration (Figure 10B, 10D) in HT29 (Figure 10A, 10B) and HCT-116 (Figure 10C, 10D) cells.

## DISCUSSION

Various tumor cells secrete exosomes but the isolation of these cancer-specific exosomes is currently a challenge due to a lack of specific markers. Previously, we observed that GPC1 may be a specific marker of exosomes in cancer patients [11, 12]. We also found that a poor prognosis and relapse in stage III colon cancer patients could be predicted by the circulating GPC1^+^ exosomes, or the regulatory microRNA of GPC1 (miR-96-5p and miR-149). This study first demonstrated that GPC1 can activate EMT and increase invasion and migration of colon cancer cells.

Almost all colorectal cancer patients that undergo curative resection will experience recurrence within four years [6]. Stage III colorectal cancer patients exhibit a higher recurrence rate than stage II patients [5]. The high cumulative recurrence rate deserves a substantial effort to identify specific biomarkers for early detection of relapse in colon cancer patients. A recent study found that GPC1 positive exosomes are a highly specific and sensitive marker suitable for early diagnosis of pancreatic cancer [12]. This study demonstrated that the percentage of GPC1^+^ plasma exosomes in total plasma exosomes measured one day before and one week after surgery was significantly higher in stage IIIC colon cancer patients than stage IIIA CRC patients. This finding suggests that GPC1^+^ plasma exosomes are associated with the severity of disease. Moreover, the percentage of GPC1^+^ plasma exosomes in total plasma exosomes was significantly higher in CRC patients with relapse than patients without relapse, in CRC patients that died with relapse than in patients that survived with relapse, in CRC patients that survived from relapse than in patients that survived without relapse, and the GPC1+ plasma exosome counts progressively increased in relapsed colon cancer patients nine months after surgery. These findings indicate that the level of GPC1^+^ plasma exosomes is a marker for the relapse and poor prognosis in patients with stage III CRC.

Our findings here suggest two major points: 1) the GPC1^+^ plasma exosome is a marker for the relapse in patients with stage III CRC, and 2) clinical management of relapse should be started before ninth months after surgery through measuring GPC1^+^ plasma exosomes. Notably, the percentage of GPC1^+^ plasma exosomes significantly decrease one week after surgery compared to the percentage of GPC1^+^ plasma exosomes one day before surgery, suggesting that circulating GPC1^+^ exosomes may be associated with circulating tumor cell count or the total tumor amount in body. Thus, the re-increase of circulating GPC1 positive exosomes in survived colon cancer patients with relapse may imply a re-population of tumor cells and formation of relapsed tumor mass locally or at a distal location. Our results also show that a short overall survival time for colon cancer patients correlated with a high percentage of GPC1^+^ plasma exosomes one day before and one week after surgery, which suggests that the circulating GPC1^+^ exosomes are a marker for poor prognosis in colon cancer patients.

The epithelial to mesenchymal transition is a complex program and has been associated with tumorigenesis, malignancy, invasion, and metastasis [22]. The decrease in E-cadherin and increase in vimentin expression are thought to be the specific markers of EMT [23]. Our results here show that overexpression of *GPC1* gene significantly decreased E-cadherin and increased vimentin protein expression in HT29 and HCT-116 cells. During EMT activation, the transdifferentiation of epithelial cells into motile mesenchymal cells needed reprogramming of gene expression, including Snail [24]. Our study found that overexpression of *GPC1* gene significantly upregulated Snail1 and Snail2 protein expression in these two CRC cell lines. Moreover, overexpression of *GPC1* gene increased invasion and migration in HT29 and HCT-116 cells while silencing of *GPC1* gene expression had the opposite effect. Therefore, the upregulation of GPC1 protein expression in exosomes may be involved in the relapse of colon cancer patients through increasing EMT and subsequent enhancing invasion and migration of colon cancer cells.

The regulatory roles of miR-96-5p and miR-149 on *GPC1* gene expression have been observed in pancreatic cancer and human endothelial cells [14, 15]. Our results have now identified that miR-96-5p and miR-149 targets the 3-UTR of human *GPC1* gene. Overexpression of miR-96-5p and miR-149 significantly inactivated EMT and inhibited invasion and migration in both HT29 and HCT-116 cells. Inhibition of these two miRNAs using inhibitors significantly increased invasion and migration in both HT29 and HCT-116 cells. These findings suggest that these two microRNAs regulate the biological functions of GPC1 protein. In this study, we also observed that low circulating miR-96-5p and miR-149 levels one day before surgery correlated with high TNM stage, short survival, poor prognosis, and relapse in stage III colon cancer patients. The levels of circulating miR-96-5p and miR-149 decreased nine months after surgery in relapsed colon cancer patients. These findings suggest that miR-96-5p and miR-149 not only are biomarkers for the disease severity, prognosis, and relapse of CRC patients, but also directly regulate the level of GPC1 positive plasma exosomes.

We conclude that a severe clinical status, poor prognosis, and relapse in stage III colon cancer patients can be suggested by observing high circulating GPC1 positive exosomes before and after surgery as well as low circulating miR-96-5p and miR-149 before surgery. We also find that GPC1 may be involved in EMT activation, invasion, and migration of colorectal cancer cells.

## MATERIALS AND METHODS

### Ethics

This study was pre-approved by the Ethics Committee of Human Study of Xiangya Hospital and written informed consent was obtained from all patients. The study was performed in accordance with the Helsinki Declaration.

### Sample collection

We collected 20 ml of peripheral fasting blood samples from 85 patients with stage III colon cancer one day before surgery and one week and three, six, nine, 12, 15, 19, 21, and 24 months after surgery. The blood samples were collected at Xiangya Hospital, Central South University between December, 2013 and December, 2015. These patients underwent surgical resection of primary tumor and regional lymph nodes, followed by FOLFOX 4 or 6 regimen of post surgery chemotherapy. No chemotherapy or radiotherapy was initiated before surgery. Among the 85 colon cancer patients, 48 were male and 37 were female with a mean age of 48.5 years. Of the 85 patients, 25 were TNM IIIA stage, 32 were IIIB, and 28 were IIIC disease. The patients were followed up for two years and survival information from all patients was collected by phone, email, and/or mail. Of the 85 patients, 24 survived less than two years, but 61 survived longer than two years. The patients were examined for relapse every six months. The information of relapse including local relapse and distal metastasis was collected. Among the 85 patients, local relapse and distal metastasis in liver, lung, bone, brain, etc. was found in 44 patients.

### Isolation and purification of exosomes

We collected 20 ml of peripheral fasting blood samples into EDTA tubes and centrifuged at 3000 × g for 5 minutes at 4°C. The plasma was collected and immediately frozen at −80°C. Exosomes were isolated from plasma using ExoCapTM Exosome Isolation and Enrichment kit (JSR Micro Materials Innovation, Sunnyvale, CA, USA) by following the manufacturer's protocol. The isolated exosomes were purified using sucrose density gradients as previously described [12]. The percentage of GPC1^+^ plasma exosomes was determined by flow cytometry. Briefly, one mg of exosomes was incubated with 300 μl of aldehyde/sulphate latex beads (Invitrogen Inc., Carlsbad, CA, USA) for 20 min at room temperature. The reaction was stopped with 100 mM glycine and 2% bovine serum albumin (BSA) in **phosphate buffered saline** (PBS) for 30 min at room temperature. The exosomes-bound beads were then washed with 1 × PBS containing 2% BSA and centrifuged for one min at 14,800g. The re-suspended beads were incubated with anti-GPC1 antibody (Santa Cruz Biotechnology, Santa Cruz, CA, USA) for 30 min, followed by incubation with Alexa-488-tagged secondary antibodies (Life Technologies, Carsbad, CA, USA) for 30 min at 4°C with rotation. Secondary antibody incubation alone was used as control and to gate the beads with GPC1-bound exosomes. The percentage of beads with GPC1^+^ exosomes was calculated.

### Western blot analysis

Cells were lysed as previously described and the homogenate was used for Western blot analysis [25]. The protein concentration was determined by a Bradford Assay (Bio-Rad Laboratories, Hercules, CA, USA). Cell homogenate containing 10 μg protein was loaded per well. After transfering, the membranes were blocked with 5% nonfat dried milk for 1 hr and incubated overnight at 4°C with antibodies for human GPC1 (sc-101827), E-cadherin, vimentin, Snail1, Snail2, and GAPDH (Santa CruzBiotechnology), and followed by incubation with horseradish peroxidase-conjugated secondary antibodies (Cell Signaling Technology, Danvers, MA, USA) for 2 hours at room temperature. The immunoreactive proteins were visualized using a chemiluminescence substrate (ECL Prime, Amersham Scienes, Pittsburgh, PA, USA). The images were taken using ChemiDoc XRS^+^ system and analyzed using Image Lab 4.1 (Bio-Rad Laboratories).

### Real-time amplification of microRNA

Total RNA was extracted from plasma using Trizol reagent (Invitrogen, Carlsbad, CA, USA) by following the user manual. Reverse transcription and real-time PCR was performed as previously described [26]. The miR-96-5p was amplified using forward primer: 5’-TTTGGCACTAGCACATTTTTGCT-3’, miR-149 was amplified using forward primer: 5’-TC TGGCTCCGTGTCTTCACTCCC-3’, and U6 was amplified using forward primer: 5’-GCTTCGGCAGCACATATACTAAAAT-3’. The reverse primers for miRNA and U6 were provided with the SYBR Premix Ex Taq II kit (TaKaRa Bio Inc, Japan). miRNA expression was quantitatively analyzed using the comparative CT method (^ΔΔ^CT). U6 mRNAs were used as internal control [26].

### Cell culture

HT29 and HCT-116 are human colon carcinoma cell lines, which were obtained from American Type Culture Collection (ATCC). 293T is a human embryonic kidney (HEK) cell line transformed with large T antigen, which was purchased from Invitrogen Inc (Carlsbad, CA, USA). All cells were cultured in DMEM medium (Invitrogen) containing 10% fetal calf serum, 100 μg/ml of streptomycin, and 100 units/ml penicillin at 37°C, 5% CO_2_.

### Preparation of adenoviruses

The miRNA expression vector was constructed according to our previously published protocol [27]. The matured miR-96-5p (5’-UUUG GCACUAGCACAUUUUUGCU-3’) and miR-149 (5’-UCUGGCUCCGUGUCUUCACUCCC-3’) sequence was used for synthesizing the minigene to express the miRNA. Briefly, the minigene was cloned into the pSilencer-3.0 vector (Ambion Inc., Austin, TX, USA). The miRNA transcription cassette including the H1 promoter, miRNA minigene, and a thymidine tail was subcloned into the pShuttle vector of pEasy-1 adenovirus packaging system. To prepare an adenovirus to express siRNA of human *GPC1* gene, the miRNA sequence was replaced with the siRNA sequence of human GPC1 (GCTGGTCTACTGTGCTCAC) [28]. To prepare adenovirus to overexpress the human GPC1 gene, the cDNA of human *GPC1* gene was amplified by PCR from a human cDNA library using forward primer: 5’-GC*GGTACC*ATGGAGCTC CGGGCCCGAGGC-3’ and reverse primer: 5’-GC*AAGCTT*GTTACCGCCACCGGGGCCTGGC-3’. The PCR product was purified, digested, and then cloned into the pShuttle vector. The adenoviruses that express GPC1 (Ad-GPC1), GPC1 siRNA (Ad-siGPC1), miR-96-5p (Ad-miR96), and miR-149 (Ad-miR149) were produced, identified, and purified as previously described [27]. As a negative control, a 22-nt sequence with no known target in the human genome was used to produce the control virus (Ad-NC) [27].

### Dual-Luciferase reporter assay

Human *GPC1* gene mRNA 3’-UTR (three prime untranslated region, 600bp, XM_011510976) containing the putative binding site of miR-96-5p and miR-149 was amplified by PCR from a human cDNA library and subcloned into pmiR-RB-REPORT (wild-type, Wt), a Firefly/Renilla luciferase reporter vector (RiboBio Co, Guangzhou, China). At the same time, the binding site of miR-96-5p or miR-149 was mutated from the cloned 3’-UTR of human *GPC1* gene by overlap PCR [29] and also subcloned into pmiR-RB-REPORT (Mut-96, Mut-149). 293T cells were seeded at 1×10^6^ cells / 10-cm dish one day before transfection and co-transfected with 800 ng of pmiR-RB-GPC1-3’UTR (Wt, Mut-96, or Mut-149) plasmid and 2μg of miR-96-5p (5’-UUUGGCACUAGCACAUUUUUGCU-3’) or miR-149 mimics (5’-UCUGGCUCCGUGUCUU CACUCCC-3’) or NC mimics (5’-UUCUCCGA ACGUGUCACGUTT-3’) using lipofectamine 2000 transfection reagent by following the manufacturer's instructions (Life Technologies, Carlsbad, CA, USA). Twenty-four hours later, firefly and renilla luciferase activities were measured using Dual-luciferase assay kit (Promega, Madison, WI, USA). The renilla luciferase signal was normalized to the firefly luciferase signal for each sample.

### Cell invasion assay

The invasion assay was performed in HT29 and HCT-116 cells using basement membrane extract (BME) according to the manufacturer's manual (Chemicon International Inc., MA Temecula, CA, USA). Briefly, cells were infected with 10 MOI (multiple of infection) of Ad-NC, Ad-GPC1, Ad-siGPC1, Ad-miR96, and Ad-miR-149 (10 MOI) virus for 12 hrs. Cells were harvested and resuspended in basal medium at 25,000 cells/well with FGF (10 ng/ml). Cells were then added on Matrigel in the 96-well plates. After 24 hours of incubation, invasion of cells towards bottom side of the well was measured using hematoxylin staining for 10 minutes after fixed with 95% alcohol for 10 min. Cells were counted under an inverted microscope.

### Cell migration assay

HT29 and HCT-116 cells were infected with 10 MOI of Ad-NC, Ad-GPC1, Ad-siGPC1, Ad-miR96, and Ad-miR-149 virus for 12 hrs. After being resuspended in basal medium, 2×10^5^ cells/well were seeded in 24-well plates containing 8.0-μm polycarbonate membrane (Chemicon International) and FGF (10 ng/ml). The cell migration assay was performed by incubating cells at 37°C, 5% CO_2_ for 24 hrs. Cells at bottom of the membranes were stained and the staining was extracted into extraction buffer. The extracted buffer was calorimetrically measured at 560 nm.

### The effects of anti-miRNA on cell invasion and migration

HT29 and HCT-116 cells (1×10^6^ cells) were seeded in 10-cm dish one day before transfection with 2μg of negative control (NC mimic inhibitor: 5’-AAACGUGACACGUUCGGAGAA-3’), miR-96-5p antisense nucleotide (5’-AGCAAAAAUGUGCUAGUGCCAAA-3’), or miR-149 antisense nucleotide (5’-GGGAGUGAAGACACGGAGCCAGA-3’) using lipofectamine 2000 transfection reagent by following the manufacturer's instructions (Life Technologies, Carlsbad, CA, USA). Twenty-four hours later, the cells were harvestedand resuspended in basal medium for invasion and migration assay as described above.

### Statistical analysis

We analyzed data using SPSS v17.0 (Chicago, IL, USA) and presented data as mean ± standard error (SEM). One-way ANOVA or two-tailed student's t-test was used for statistical analyses of gene expression data. The overall survival of patients with colon cancer was analyzed using Kaplan-Meier univariate survival analysis and log-rank tests. A *p*<0.05 was considered statistically significant.

## References

[1] Raskov H, Pommergaard HC, Burcharth J, Rosenberg J (2014). Colorectal carcinogenesis--update and perspectives. World J Gastroenterol.

[2] Jemal A, Center MM, Ward E, Thun MJ (2009). Cancer occurrence. Methods Mol Biol.

[3] Bingham SA, Day NE, Luben R, Ferrari P, Slimani N, Norat T, Clavel-Chapelon F, Kesse E, Nieters A, Boeing H, Tjønneland A, Overvad K, Martinez C (2003). Dietary fibre in food and protection against colorectal cancer in the European Prospective Investigation into Cancer and Nutrition (EPIC): an observational study. Lancet.

[4] van de Velde CJ, Boelens PG, Tanis PJ, Espin E, Mroczkowski P, Naredi P, Pahlman L, Ortiz H, Rutten HJ, Breugom AJ, Smith JJ, Wibe A, Wiggers T (2014). Experts reviews of the multidisciplinary consensus conference colon and rectal cancer 2012: science, opinions and experiences from the experts of surgery. Eur J Surg Oncol.

[5] Tsikitis VL, Larson DW, Huebner M, Lohse CM, Thompson PA (2014). Predictors of recurrence free survival for patients with stage II and III colon cancer. BMC Cancer.

[6] Sadahiro S, Suzuki T, Ishikawa K, Nakamura T, Tanaka Y, Masuda T, Mukoyama S, Yasuda S, Tajima T, Makuuchi H, Murayama C (2003). Recurrence patterns after curative resection of colorectal cancer in patients followed for a minimum of ten years. Hepatogastroenterology.

[7] Aikawa T, Whipple CA, Lopez ME, Gunn J, Young A, Lander AD, Korc M (2008). Glypican-1 modulates the angiogenic and metastatic potential of human and mouse cancer cells. J Clin Invest.

[8] Matsuda K, Maruyama H, Guo F, Kleeff J, Itakura J, Matsumoto Y, Lander AD, Korc M (2001). Glypican-1 is overexpressed in human breast cancer and modulates the mitogenic effects of multiple heparin-binding growth factors in breast cancer cells. Cancer Res.

[9] Su G, Meyer K, Nandini CD, Qiao D, Salamat S, Friedl A (2006). Glypican-1 is frequently overexpressed in human gliomas and enhances FGF-2 signaling in glioma cells. Am J Pathol.

[10] De Robertis M, Arigoni M, Loiacono L, Riccardo F, Calogero RA, Feodorova Y, Tashkova D, Belovejdov V, Sarafian V, Cavallo F, Signori E (2015). Novel insights into Notum and glypicans regulation in colorectal cancer. Oncotarget.

[11] Li J, Chen Y, Guo X, Zhou L, Jia Z, Peng Z, Tang Y, Liu W, Zhu B, Wang L, Ren C (2017). GPC1 exosome and its regulatory miRNAs are specific markers for the detection and target therapy of colorectal cancer. J Cell Mol Med.

[12] Melo SA, Luecke LB, Kahlert C, Fernandez AF, Gammon ST, Kaye J, LeBleu VS, Mittendorf EA, Weitz J, Rahbari N, Reissfelder C, Pilarsky C, Fraga MF (2015). Glypican-1 identifies cancer exosomes and detects early pancreatic cancer. Nature.

[13] Zhang Z, Coomans C, David G (2001). Membrane heparan sulfate proteoglycan-supported FGF2-FGFR1 signaling: evidence in support of the “cooperative end structures” model. J Biol Chem.

[14] Li C, Du X, Tai S, Zhong X, Wang Z, Hu Z, Zhang L, Kang P, Ji D, Jiang X, Zhou Q, Wan M, Jiang G (2014). GPC1 regulated by miR-96-5p, rather than miR-182-5p, in inhibition of pancreatic carcinoma cell proliferation. Int J Mol Sci.

[15] Chamorro-Jorganes A, Araldi E, Rotllan N, Cirera-Salinas D, Suárez Y (2014). Autoregulation of glypican-1 by intronic microRNA-149 fine tunes the angiogenic response to FGF2 in human endothelial cells. J Cell Sci.

[16] Nikitovic D, Kouvidi K, Voudouri K, Berdiaki A, Karousou E, Passi A, Tzanakakis GN (2014). The motile breast cancer phenotype roles of proteoglycans/glycosaminoglycans. Biomed Res Int.

[17] Bosukonda A, Carlson WD (2017). Harnessing the BMP signaling pathway to control the formation of cancer stem cells by effects on epithelial-to-mesenchymal transition. Biochem Soc Trans.

[18] Lee JG, Ko MK, Kay EP (2012). Endothelial mesenchymal transformation mediated by IL-1β-induced FGF-2 in corneal endothelial cells. Exp Eye Res.

[19] Ding K, Lopez-Burks M, Sánchez-Duran JA, Korc M, Lander AD (2005). Growth factor-induced shedding of syndecan-1 confers glypican-1 dependence on mitogenic responses of cancer cells. J Cell Biol.

[20] Götte M, Kersting C, Radke I, Kiesel L, Wülfing P (2007). An expression signature of syndecan-1 (CD138), E-cadherin and c-met is associated with factors of angiogenesis and lymphangiogenesis in ductal breast carcinoma in situ. Breast Cancer Res.

[21] Scanlon CS, Van Tubergen EA, Inglehart RC, D’Silva NJ (2013). Biomarkers of epithelial-mesenchymal transition in squamous cell carcinoma. J Dent Res.

[22] Felipe Lima J, Nofech-Mozes S, Bayani J, Bartlett JM EMT in Breast Carcinoma-A Review. J Clin Med.

[23] Kim YS, Yi BR, Kim NH, Choi KC (2014). Role of the epithelial-mesenchymal transition and its effects on embryonic stem cells. Exp Mol Med.

[24] Lamouille S, Xu J, Derynck R (2014). Molecular mechanisms of epithelial-mesenchymal transition. Nat Rev Mol Cell Biol.

[25] Li J, Chen Y, Guo X, Zhou L, Jia Z, Tang Y, Lin L, Liu W, Ren C (2016). Inhibition of miR-15b decreases cell migration and metastasis in colorectal cancer. Tumour Biol.

[26] Zhang Y, Wang Y, Wang L, Bai M, Zhang X, Zhu X (2015). Dopamine Receptor D2 and Associated microRNAs Are Involved in Stress Susceptibility and Resistance to Escitalopram Treatment. Int J Neuropsychopharmacol.

[27] Li J, Zhang Y, Zhao J, Kong F, Chen Y (2011). Overexpression of miR-22 reverses paclitaxel-induced chemoresistance through activation of PTEN signaling in p53-mutated colon cancer cells. Mol Cell Biochem.

[28] Belting M, Mani K, Jönsson M, Cheng F, Sandgren S, Jonsson S, Ding K, Delcros JG, Fransson LA (2003). Glypican-1 is a vehicle for polyamine uptake in mammalian cells: a pivotal role for nitrosothiol-derived nitric oxide. J Biol Chem.

[29] Wei H, Hu J, Wang L, Xu F, Wang S (2012). Rapid gene splicing and multi-sited mutagenesis by one-step overlap extension polymerase chain reaction. Anal. Biochem.

